# Polyclonal Aptamer Libraries from a FluRoot-SELEX for the Specific Labeling of the Apical and Elongation/Differentiation Zones of *Arabidopsis thaliana* Roots

**DOI:** 10.3390/ijms232012220

**Published:** 2022-10-13

**Authors:** Ann-Kathrin Kissmann, Dennis Wolf, Markus Krämer, Franziska Müller, Valerie Amann, Hu Xing, Kay-Eberhard Gottschalk, Tanja Weil, Ruth Eichmann, Patrick Schäfer, Frank Rosenau

**Affiliations:** 1 Institute of Pharmaceutical Biotechnology, Ulm University, Albert-Einstein-Allee 11, 89081 Ulm, Germany; 2 Max-Planck-Institute for Polymer Research Mainz, Ackermannweg 10, 55128 Mainz, Germany; 3 Institute of Experimental Physics, Ulm University, Albert-Einstein-Allee 11, 89081 Ulm, Germany; 4 Institute of Phytopathology, Research Centre for BioSystems, Land Use and Nutrition, Justus Liebig University, 35392 Giessen, Germany

**Keywords:** *Arabidopsis thaliana*, aptamer library, cell wall, FluRoot-SELEX, root development

## Abstract

In more than 30 years of aptamer research, it has become widely accepted that aptamers are fascinating binding molecules for a vast variety of applications. However, the majority of targets have been proteins, although special variants of the so-called SELEX process for the molecular evolution of specific aptamers have also been developed, allowing for the targeting of small molecules as well as larger structures such as cells and even cellular networks of human (tumor) tissues. Although the provocative thesis is widely accepted in the field, that is, in principle, any level of complexity for SELEX targets is possible, the number of studies on whole organs or at least parts of them is limited. To pioneer this thesis, and based on our FluCell-SELEX process, here, we have developed polyclonal aptamer libraries against apices and the elongation/differentiation zones of plant roots as examples of organs. We show that dedicated libraries can specifically label the respective parts of the root, allowing us to distinguish them in fluorescence microscopy. We consider this achievement to be an initial but important evidence for the robustness of this SELEX variant. These libraries may be valuable tools for plant research and a promising starting point for the isolation of more specific individual aptamers directed against root-specific epitopes.

## 1. Introduction

**Scheme 1:**
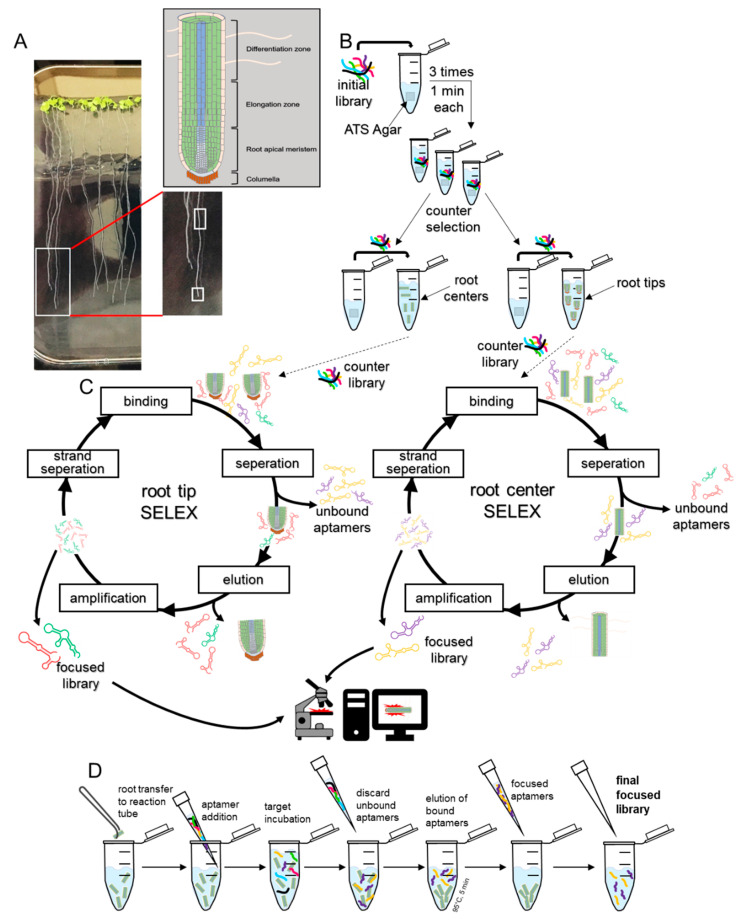
FluRoot-SELEX-based evolution of focused aptamer libraries for the specific detection of *A. thaliana* root zones. (**A**) *A. thaliana* growth on vertical square Petri dishes in ATS medium and schematic overview of root developmental zones. (**B**) Initial counter-selection via the incubation of an initial aptamer library (~6 × 10^13^ individual aptamers) containing forty randomized nucleotides flanked by two primer binding sites (23 nt each), first with ATS agar cubes (threefold counter-selection) and then with counter root zone segments to gain aptamer “counter libraries” with reduced sequence spaces. (**C**) Specific polyclonal aptamer libraries selected by SELEX against root tips (containing apical meristem and elongation zone) and centers (containing differentiation zone without lateral roots) via the reduction of sequence diversity by incubating the counter-selected aptamer libraries with the target sections. Aptamers that had an appropriate three-dimensional structure bonded to the target cell walls, and the remaining unbound aptamers were subsequently removed. Bound aptamers were then eluted from the target root section, amplified by PCR, and the undesired complementary strands were removed prior to the next selection round. Using fluorescence microscopy, specific target detection for the binding of focused aptamer libraries to root tips and root centers was conducted. (**D**) Experimental set-up for aptamer incubation with root tips or root centers.

Since their introduction a few decades ago, single-stranded oligonucleotides such as RNA or ssDNA have emerged as new alternatives to antibodies, with surprisingly high affinity and specificity as well as chemical and physical stability, combined with overall low immunogenicity [1]. They are even considered nucleotide analogs and, hence, termed “chemical antibodies” [2]. These binding entities can be evolved and isolated from large random sequence libraries in the iterative selection process, carried out completely in vitro, a process called the Systematic Evolution of Ligands by EXponential enrichment (SELEX) [3,4]. In repeated rounds of target binding and PCR-mediated amplification, high-affinity aptamers can be selected specifically against an enormous variety of targets, as they can acquire different secondary and tertiary structures [5]. This targeted selection technology has been frequently modified and, thus, improved to develop more efficient, time-saving, and target-specialized SELEX strategies to allow for the selection of aptamers with specific binding characteristics for different molecules in numerous applications. Nowadays, adapted SELEX processes such as Capture-SELEX or FluMag-SELEX enable the selection of aptamers for small solute molecules [6], chemical compounds such as metal ions [7], as well as proteins and their cofactors [8,9,10]. To obtain aptamers that are specific towards whole cells and certain microorganisms, Cell-SELEX and FluCell-SELEX have been developed to target intact living cells such as the pathogenic bacterium *Pseudomonas aeruginosa* or different mammalian cells, such as cancer cells, without knowing the exact membrane target in advance [11,12,13,14,15,16,17]. Even against complex consortia of cells such as tumors, specialized technologies allow for the selection of highly specific aptamers to work on pathological tissues [18,19]. Thus, these binding molecules cannot only be used in various fields, such as biomarker discovery [20], electronic biosensing [21], diagnostics [22], and drug delivery [23], and they cannot be used as pharmaceutical compounds in molecular therapy [24,25]; however, they are also attractive molecules for deciphering cellular processes via molecular imaging and labeling applications [26]. Even though aptamers are commonly used in biomedical research and environmental monitoring [27,28], their daily use has not yet been found in the field of cell wall research [29,30]. Therefore, to specifically label and distinguish between the growth zones of the roots of the model organism *Arabidopsis thaliana*, we developed a selection strategy to evolve aptamer libraries using Fluorescence Root-SELEX (“FluRoot-SELEX”) (Scheme 1). Based on the usage of fluorescently labeled aptamers throughout the complete selection process, naturally immobilized targets on the surface of *A. thaliana* root cell walls were targeted and labeled by oligonucleotides. Plant roots are not only essential for anchoring the plants, but also for overall plant health and development, as roots absorb water and nutrients. Coping with varying functions, particularly under acute stress conditions, changing environments, and the influence of different soil organisms, led to the formation of highly complex tissues with defined root developmental zones along the longitudinal root axis, which exhibit different functions [31,32,33]. The various developmental zones are the result of cell differentiation starting at the apical meristem, where cell division takes place [34,35]. In the axial dimension, the root is organized in concentrically arranged cell types, with the vascular tissue at its center.

Here, in a differential selection process, cell wall-targeted molecular probes are evolved that are capable of distinguishing between these root zones, which include the root apical meristem with the columella and the elongation zone (“root tips”), and segments consisting of the differentiation zone (“root centers”) (Scheme 1A). For the FluRoot-SELEX process, plants were grown on vertical square Petri dishes on ATS medium (Scheme 1A), and *A. thaliana* root zones were harvested and subjected to the selection process (Scheme 1C), where, first, an initial counter-selection (Scheme 1B) against the ATS medium and against the counter root zone segments took place in order to generate aptamer “counter libraries” with reduced sequence spaces and, hence, high specificity for cell walls of key root zones.

With the significant simplification of aptamer textbook methods, we recently showed that, without the canonical isolation of individual aptamers, already, the focused polyclonal libraries are able to outperform single aptamers and, therefore, can be used after a sufficient level of enrichment in various applications [10,12]. Therefore, we can anticipate that using focused polyclonal libraries will be beneficial due to greater precision as well as the larger sequence space available for highly efficient target recognition and enhanced performance. Such focused aptamer libraries were evolved in the new FluRoot-SELEX process in a total of seven rounds of selection using complex consortia of cells, such as plant root developmental zones, without knowing the exact targets in advance to serve as monitoring molecules on *A. thaliana* root surfaces.

## 2. Results

**Figure 1:**
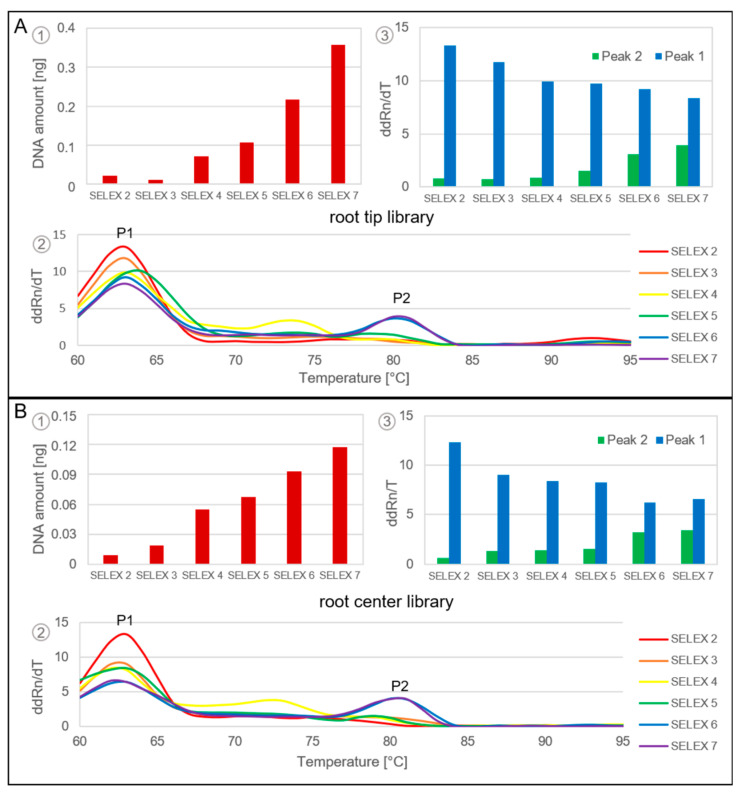
Real-time PCR-based evolution analysis of the (**A**) root tip and (**B**) root center aptamer libraries of rounds two through seven. (1) DNA concentrations in eluates immediately after the SELEX rounds, (2) melting curves with peak 1 (P1) and peak 2 (P2) as the two major temperature peaks observed in the experiments, and (3) peak-shifting analyses for P1 and P2. All experiments were conducted in triplicates (*N* = 3) using qTOWER^3^G Touch (Analytik Jena GmbH, Jena, Germany).

With the overall aim of evolving focused aptamer libraries, two independent FluRoot-SELEX processes were carried out simultaneously in order to generate a tool to differentiate between the growth zones of *A. thaliana* roots. In a total of seven selection rounds, the specificity toward the dedicated root developmental zones—the root tip (columella, root apical meristem, elongation zone) and root center (differentiation zone without lateral roots)—was remarkably increased. Counter-selections prior to every selection step led to an enhanced stringency and raised selection pressure, thus reducing non-specific aptamers. The respective libraries of each SELEX round underwent an evolutionary process with characteristic consequences for the composition of the individual sequences, as well as for the sequence space, where a tendency toward a higher GC-content in the molecules is typically introduced [12,13]. This tendency toward higher GC-content in the molecules is accompanied by an increase in melting temperatures in PCR reactions since guanine−cytosine (GC) bonding is based on three hydrogen bonds, whereas adenine−thymine (AT) pairing relies only on two hydrogen bonds [36]. First, we analyzed the final DNA concentrations of the eluted aptamer libraries immediately after the SELEX rounds, which was the highest in the final round (seven) for both the root tip and root center aptamers (Figure 1(A1,B1)). The increase in GC-content in the molecules was then analyzed using the increase in melting temperatures in quantitative PCR reactions, which we tentatively tested in all SELEX rounds, and, in fact, two major peaks occurred for the melting temperatures 63 °C and 81 °C (peak 1 and peak 2; P1 and P2) in the real-time PCR analyses (Figure 1(A2,B2)). Between rounds two and seven, the relative fluorescence intensity (ddRn/dT-value) dropped for peak 1 and simultaneously increased for peak 2, indicating the expected shift toward higher GC-content in both libraries (Figure 1(A2,B2)). This peak-shifting was further analyzed for the level of decrease for peak 1, as well as the level of increase for peak 2 (Figure 1(A3,B3)). For root tip aptamer libraries, peak 1 decreased over rounds two through seven by 37%, whereas peak 2 increased by 79% (Figure 1(A3)). Peak 1 of the root center libraries decreased by 47% over the SELEX process, and for peak 2, an increase of 83% was observed (Figure 1(B3)).

**Figure 2:**
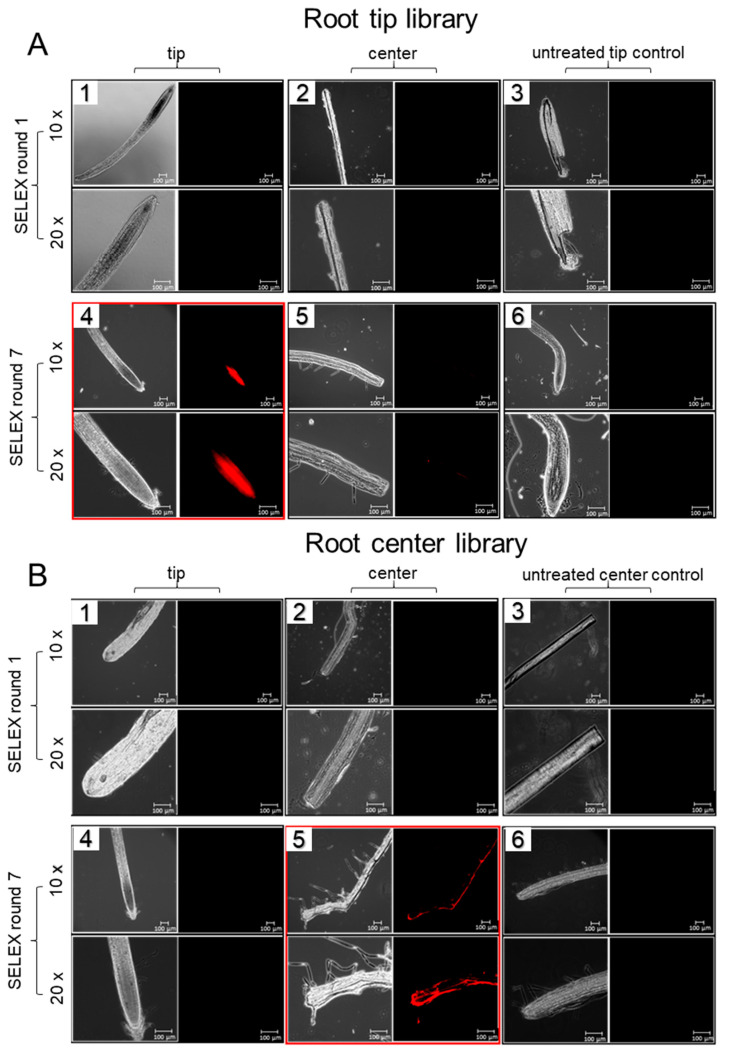
Visualization of the specific fluorescence labeling of *A. thaliana* root zones by Cy5-labeled root tip and root center aptamer libraries (excitation: 635 nm) using fluorescence and light-transmitting filters at 10×/20× magnitudes. In the process, the aptamers were added to the dedicated and opposite target sites, and, furthermore, the targets without aptamer treatments are shown as controls. (**A**) The target sites with root tip aptamers are displayed, and in (**B**), those with root center aptamers are shown. The upper panel shows the libraries of the first FluRoot-SELEX round and the lower panel represents the seventh round. Plants used for the analyses were cultivated for 12–14 days, and all experiments were performed using 10 pmol aptamers. Microscopy was performed using a Leica DMi8 coded (Leica Microsystems CMS GmbH, Wetzlar, Germany) under transmitted light and using the Y5 filter (excitation: 590–650 nm and emission: 662–738 nm) for fluorescence imaging.

In the next step, the highly specific and selective binding of the aptamer libraries was validated with fluorescence microscopy using aptamers labeled with cyanine 5 (Cy5) after amplification with dye-functionalized forward primers. For this, 10 pmol of each focused polyclonal aptamer library was incubated for 60 min with the dedicated targets, as well as the counter root zone segments, and subsequently visualized using both transmitted light and fluorescence filters. To monitor the evolution process of the aptamer specificity over the whole FluRoot-SELEX process, the final aptamer libraries were compared with the aptamer libraries isolated from the first selection round. As expected, the final root tip-specific aptamer library efficiently labeled the dedicated target root zone segment (Figure 2(A4)); whereas, the first-round aptamers did not deliver any red fluorescence signal (Figure 2(A1)). Similarly, no fluorescence signals were detected after incubating the first-round root tip library with the non-target root center segments (Figure 2(A2)). As intended, the final root tip library resulted in only marginal signals with the root center segments (Figure 2(A5)) as the control target, thus proving that, during the evolution process of root center-specific aptamers, they were not enriched in considerable quantities. To exclude the possibility that the observed signals were false positives resulting from potentially existing autofluorescence in the plant tissues, solely untreated root tips (Figure 2(A3,A6)) and root centers (Figure 2(B3,B6)) were microscopically analyzed as negative controls. Comparable results were obtained with the root center-specific final library, which could perfectly label its intended target (Figure 2(B5)) but completely failed in labeling root tips (Figure 2(B4)) as the opposite root zone segment in our study. As seen in the SELEX evolution of the root tip libraries (round 1 to round 7), the first SELEX round of root center aptamers was not efficient enough in labeling root zone segments (Figure 2(B1,B2)) to deliver convincing visual fluorescence signals.

**Figure 3:**
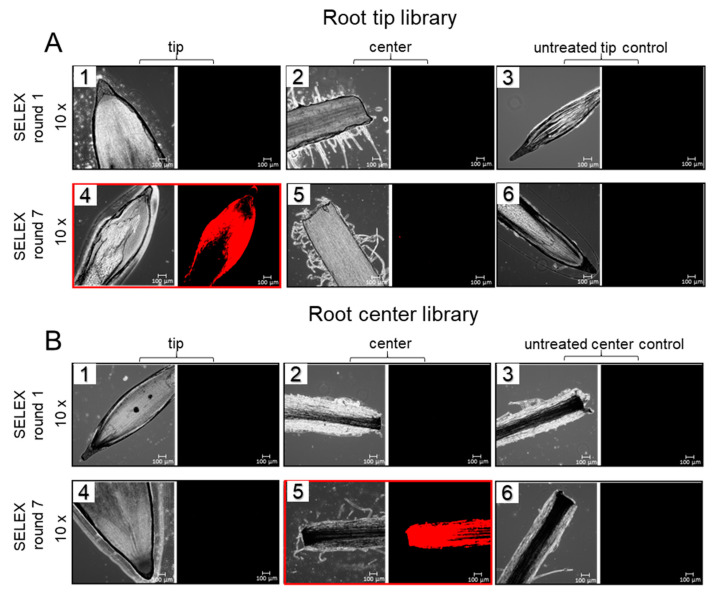
Visualization of the specific fluorescence labeling of the root developmental zones of *H. vulgare* L. by Cy5-labeled root tips and root center aptamer libraries (excitation: 635 nm) using fluorescence and light0transmitting filters at a 10× magnitude. In the process, the aptamers were added to the desired and opposite target sites, and, furthermore, the targets without aptamer treatment are shown as controls. (**A**) The target sites with root tip aptamers are displayed, and in (**B**), those with root center aptamers are shown. The upper panel shows the libraries of the first FluRoot-SELEX round and the lower panel represents the seventh round. The plants used for the experiment were cultivated for 3 days, and 10 pmol of aptamers were used. Microscopy was performed using a Leica DMi8 coded (Leica Microsystems CMS GmbH, Wetzlar, Germany) under transmitted light and using the Y5 filter (excitation: 590–650 nm and emission: 662–738 nm) for fluorescence imaging.

Both the root tip and root center libraries were suited to specifically label the dedicated developmental zones of *A. thaliana* roots for fluorescence microscopy. We decided to test whether this would also hold true for the root developmental zones of the monocotyledonous plant barley (*Hordeum vulgare* L). Furthermore, for the root zones of this plant, the libraries showed remarkable specificity for both the root tip and root center without any labeling activity against the opposite root zone segment type (Figure 3). As observed before with *A. thaliana*, the round one libraries completely failed to label the designated root zones (Figure 3(A,B1,B2)), but the final, round seven libraries were fully functional (Figure 3(A,B4,B5)).

## 3. Discussion

The SELEX process for the molecular evolution of nucleic acid-based binding entities as well as the aptamers resulting from such SELEX processes have both proven in the last decades to be powerful methods and robust ligands for a vast number of different applications for an enormous variety of (molecular) targets. Special variants of SELEX exist for whole cells [11,12,13,14] and tissues, as they are relevant, especially for the isolation of aptamers for cancer diagnostics [18,19]. There, an individual aptamer that efficiently recognizes cancerous liver tissues by binding to intracellular components in the nucleus was identified [18]. However, to the best of our knowledge, aptamers directed against plant-derived targets are relatively rare, with the recent exception of aptamers developed against celluloses, which are intended as molecular tools for the analysis of cell wall components [30,37]. However, no intact cell walls/plant tissues have been used previously for the in vitro selection of aptamers specific to cellulose, only the free-standing purified target molecule itself has been used [37]. However, the use of dedicated binding molecules is of interest in molecular plant sciences, as can be deduced from the fact that antibodies have successfully been used for studying microarchitectures in root hairs, and they recognize different carbohydrate epitopes present in plant cell wall polysaccharides to locate these epitopes in the roots of developing *A. thaliana* seedlings [38,39]. Here, we adopted the established protocols for FluCell-SELEX [12,13,14] and FluMag-SELEX [9] and developed the FluRoot-SELEX process to generate polyclonal DNA aptamer libraries directed toward distinct plant tissues, in this case, the root tips of *A. thaliana* roots and the adjacent root center zone. Without knowing the exact target of the cell wall, this modified SELEX strategy allows for the identification of polyclonal aptamers against complex plant tissues. The use of polyclonal aptamer libraries is of certain interest, as it has been previously described that polyclonal aptamers can even outperform individual aptamers in labeling difficult and highly complex targets, such as whole cells [12,13]. In only seven rounds of the FluRoot-SELEX process, including counter-selections and extremely harsh selective pressure, starting with the initial library before the first selection round, we achieved a reasonable specificity. A shift toward higher melting temperatures in the aptamers’ states an elevated GC-content, which is indicative of the evolutional processes occurring during the SELEX process, resulting in drastically different sequences in the final, round seven libraries [12,13]. These resulting libraries were perfectly suited to label their dedicated target root zone without an unspecific background, thus allowing for the easy and efficient discrimination of the root zone in fluorescence microscopy. Interestingly, the libraries were also able to specifically label their target root zone not only for *A. thaliana* but also for the monocotyledonous plant *H. vulgare* L. Without yet having taken the next logical experimental path, i.e., the next-generation sequencing of the libraries and the isolation of individual aptamers via bioinformatic analyses and their characterization, the interspecies-specific cross-reactivity suggests that dicotyledonous and monocotyledonous plants share molecular target epitopes on the surfaces of their roots. In general, the target binding of aptamers is mainly achieved via structural compatibility, as well as electrostatic and van der Waals interactions and hydrogen bonding [8,40,41]. Complex targets such as root differentiation zones are represented by a consortium of various components where the cell walls of root cells mainly consist of cellulose, hemicelluloses, and pectins and undergo alterations during growth and development. It is, therefore, expected that younger dividing cells in the meristematic and transition zone at the root tip differ from older, more rigid cells with specialized functions in the elongation and differentiation zones. The polysaccharide content and composition of root cell walls especially change in the course of root development. During cell differentiation, cells expand, and cell walls undergo remodeling. Cell wall loosening during cell expansion in the elongation zone requires specific enzymes (e.g., EXPANSINs, hydrolases, peroxidases). Furthermore, secondary cell wall formation, including cell wall cross-linking and lignin deposition, provides rigidity and stability to the differentiating cell, and, again, these processes are regulated by a diversity of cell wall-modifying enzymes [42]. Previously, a carbohydrate epitope in the pectic polysaccharide rhamnogalacturonan I was detected only in the mature parts of roots using monoclonal antibodies [38]. Whether polysaccharide structures, lignin molecules, or specific proteins determine the binding of differential aptamers to younger and older root parts remains unknown. The identification of such molecular targets present on the cell wall is of definite common interest not only for aptamer research but also may offer opportunities to specifically bind and block or support surface architectures on the root surface for functional root cell wall studies. The root surface is where interactions occur in nature with the abiotic and biotic environment of the plant in the soil, including contact with beneficial or pathogenic microorganisms, which colonize roots and, hence, affect the welfare of the plant. Thus, we believe that the polyclonal libraries presented here may not only be of interest for simple labeling and the visualization of root parts but may also inspire aptamer researchers and plant scientists to attempt to use complex targets such as root developmental zones, which were used here successfully. Prospectively, aptamers against root epitopes may be on a longer timeline and may open new avenues to modify plant–microbe interactions with the aim of engineering these consortia of organisms for the optimization of agricultural processes.

## 4. Materials and Methods

### 4.1. Materials

Agarose, boric acid, isopropanol, and tris were obtained from Roth (Carl Roth GmbH and Co. KG, Karlsruhe, Germany). Bovine serum albumin, ethanol, and tRNA were purchased from Sigma-Aldrich (St. Louis, MI, USA). EDTA and Dulbecco’s Phosphate-Buffered Saline (DPBS) (1x) were ordered from Thermo Fisher Scientific (Waltham, MA, USA).

### 4.2. Plant Growth Conditions

*A. thaliana* Columbia-0 (Col-0) seeds were obtained from the Nottingham *Arabidopsis* Stock Center. Seeds were surface-sterilized and plants were grown in ATS medium [43] supplemented with 4.5 g l^−1^ Gelrite (Duchefa Biochemie) in a 23 °C day/18 °C night cycle (10 h light) at 70 μmol m^−2^s^−1^ in vertical squared Petri dishes. The cultivation time was between 10–12 days. “Root tip” samples consisted of 2–3 mm-long segments of the root tip cut with a razor blade, while “root centers” were 5 mm-long segments that were harvested 1.5–2 cm away from the root tip in the basal direction.

*Barley* (*Hordeum vulgare* L.) cultivar “Golden Promise” seeds were surface-sterilized and husks were removed. Seeds were transferred to ATS medium in a 23 °C day/18 °C night cycle in vertical squared Petri dishes covered in aluminum foil to prevent light penetration. Root segments were harvested after 3 days of cultivation. About 3 mm-long segments of the root tip were cut with a razor blade and collected as “root tip” samples, while “root centers” were 5 mm-long segments that were harvested about 2.5 cm away from the root tip in the basal direction.

### 4.3. In Vitro Selection of Aptamer Libraries against Root Tip and Elongation/Differentiation Zones

For the FluRoot-SELEX process, a commercial aptamer library (TriLink BioTechnologies, Inc, San Diego, CA, USA) containing approx. 6 × 10^13^ individual aptamers with a central random 40- nucleotide (nt) region flanked on both sides by 23 nt primer binding sites was used. The amplification and concomitant selection labeling was carried out using primers with the following sequences: cyanine 5-labeled forward primer (Cy5-FW): 5′-[Cy 5]T AGG GAA GAG AAG GAC ATA TGA T-3′ and phosphate-labeled reverse primer (P-RV): 5′-[P]T CAA GTG GTC ATG TAC TAG TCA A-3′ (Eurofins Genomics, Ebersberg, Germany). For the subsequent binding, the aptamers had to be activated according to the following scheme: first, the ssDNA library was heated to 95 °C for 5 min; afterward, it was cooled in ice for 5 min and finally incubated at 25 °C for 20 min. Counter-selections were performed with the opposite root zone segment prior to each round of selection (root tip aptamers on root centers and root center aptamers on root tips). *A. thaliana* was cultivated and harvested at 12–14 days of age. Subsequently, 5 approx. 3–5 mm-long root tips and centers of each were cut and transferred with tweezers to PCR tubes containing 20 µL DPBS buffer and were temporarily stored at 4 °C. Prior to each SELEX round, the root zone segments were washed two times with 1x DPBS. In the first round of selection, 0.1 nmol of the random ssDNA library was diluted in 200 µL DPBS and added to three different DPBS-saturated ATS-medium blocks, each for 1 min to undergo unspecific binding. The supernatant, containing unbound aptamers, was added to each root target and incubated at 25 °C for 30 min under rotational and darkened conditions. Afterward, 600 pmol of BSA (10 mg/mL) and 600 pmol tRNA (10 mg/mL) were added to the unbound aptamers and transferred to the dedicated target sites, either the root tips or root centers. After incubation for 60 min, the root zone segments were washed with 200 µL of DPBS via pipetting to remove unbound aptamers. To elute the bound aptamers, the root zone segments were heated to 95 °C in 120 µL DPBS for 5 min, and the liquid phase was removed and collected for the next round of selection. After each SELEX round, the eluted ssDNA aptamers were amplified by polymerase chain reaction (PCR). Therefore, 722.5 µL Water HPLC Plus, 200 µL 5x Herculase II reaction buffer, 12.5 µL dNTPs (10 mM, 2.5 mM each), 2.5 µL Herculase II Fusion DNA Polymerase (Agilent Technologies, Santa Clara, CA, USA), 1.25 µL Cy5-FW, and 1.25 µL P-RV were mixed on a Vortex-Genie 2 (Scientific Industries, Inc., Bohemia, NY, USA). Then, 20 µL ssDNA aptamers were added to the reaction mixture, and aliquots with 48 µL per PCR reaction tube were produced. The amplification conditions were 2 min at 80 °C, 2 min at 85 °C, 2 min at 90 °C, and 3 min at 94 °C and 25 cycles of 30 sec at 94 C, 30 sec at 56 °C, and 15 sec at 72 °C and then 2 min at 72 °C after the last cycle. Afterward, the amplicons were purified using the NucleoSpin Gel and PCR Clean-Up Kit (MACHEREY-NAGEL, Düren, Deutschland) and analyzed on a 2% agarose gel in 0.5x TBE buffer using agarose gel electrophoresis. To separate the relevant forward strand of the double-stranded PCR products, the digestion of undesired phosphorylated reverse strands using lambda exonuclease (New England Biolabs, Ipswich, MA, USA) was carried out according to the manufacturer [44]. In brief, the reaction setup included up to 5 μg dsDNA, 5 µL Lambda Exonuclease Reaction Buffer (10X), 1 µL Lambda Exonuclease, and up to 50 µL Water HPLC Plus. The reaction mix was incubated at 37 °C for 30 min. Subsequently, the reaction was stopped by adding 10 mM EDTA and a heat inactivation at 80 °C for 10 min. Afterward, the ssDNA aptamers are purified with the NucleoSpin Gel and PCR Clean-Up Kit. To quantify the amount of generated ssDNA, the NanoPhotometer^®^ NP80 (IMPLEN, Munich, Germany) was used. After the first selection round, 5 pmol ssDNA aptamers from the previous round were used in SELEX rounds 2–7. All subsequent selection rounds started with a counter-selection step using the opposite target root zone segments. Additionally, tRNA and BSA concentrations were increased throughout all rounds (+300 pmol per round), as well as during the washing steps after the final selection step (+1 washing step per round).

### 4.4. Real-Rime PCR

For an evolutionary analysis of tip and center aptamer libraries, a quantitative PCR (qPCR) was performed with the eluates of SELEX rounds two to seven using qTOWER^3^G Touch (Analytik Jena GmbH, Jena, Germany) with SYBR green I (final concentration 0.5x) (Sigma-Aldrich, St. Louis, USA). For absolute quantification, a standard curve in the range of 0.0001–1 ng was prepared with a synthetic aptamer library. The Ct values were calculated using qPCRsoft 4.0, and, thus, the DNA amount of the SELEX eluates was calculated. In addition, melting curve and peak-shifting analyses were performed.

### 4.5. Fluorescence Microscopy

Fluorescence microscopy provided the opportunity to visualize and analyze the specific target binding sites of both the final aptamer libraries. Therefore, 5 pmol from each aptamer library was activated according to the prior description and incubated with either the root center or tip for 60 min at room temperature in darkened conditions on a rotator [10,12,13,14,15,16,17]. Thereafter, the root zone segments were washed two times using 100 µL DPBS and were prepared on a slide with a glass cover. Microscopy was performed using a Leica DMi8 coded (Leica Microsystems CMS GmbH, Wetzlar, Germany) at x10 and x20 magnitude under transmitted light for phase contrast imaging and using the Y5 filter (excitation: 590–650 nm and emission: 662–738 nm) for fluorescence imaging.

## 5. Conclusions

In summary, we provided the first polyclonal aptamer libraries with specificity toward different root segments of *A. thaliana*, and we described the possibility of efficiently using these libraries in fluorescence microscopy to distinguish between root tips and root center segments. Aptamers were evolved in seven rounds of SELEX against tips and center segments, and we demonstrated that these libraries were functional for the intended application without the typically time-consuming and expensive sequencing, bioinformatic analyses, selection of individual sequences, and in-depth biochemical characterization of the resulting individual aptamers prior to application testing. This successful use of polyclonal libraries was demonstrated earlier for the detection of pathogenic and probiotic bacteria and sequencing, as the first step in the standard procedure, delivered upon bioinformatic analyses ≤10 individual aptamers, which then performed similar or worse than the respective libraries. Typically, these sequencing experiments resulted in up to several thousand individual sequences, which were then clustered in bioinformatic analyses to obtain the mentioned sequences, which then had to be synthesized and characterized experimentally to deliver a reliable statement regarding their performance and specificity. However, being already functional binding entities for labeling and visualization techniques, the *A. thaliana* libraries presented here will be subjected to complete analysis procedures, including sequencing, bioinformatic analyses, and biochemical characterization in our next study. This will subsequently allow us to identify molecular targets present on the cell wall. This detailed description of sequences already present in our *A. thaliana* libraries will hopefully inspire not only our future work but also other scientists in the field to consider enriched individual aptamer sequences for labeling procedures in their projects. This may also open new avenues to new applications, which may require specifically supporting or blocking root architectures on plant surfaces in functional cell wall studies to analyze natural interactions with the biotic and abiotic soil environment with respect to interactions between beneficial or even pathogenic microorganisms.

## Data Availability

Not applicable.
